# Traveling Letter from the Senior Editor

**Published:** 1847-11

**Authors:** 


// THE WESTERN JOURNAL.

NEW SERIES.—VOL. VIII.—NO. V.

LOUISVILLE, NOVEMBER 1, 1847.

### TRAVELING LETTERS FROM THE SENIOR EDITOR.

NUMBER FIVE.

TORONTO, September 13, 1847.

*To My Colleagues:*

GENTLEMEN: Since my last, closed at Montreal ten days ago, the difficulties I have encountered in procuring opportunities for conversation with the physicians of that city, Prescott, Kingston, and this place, have interfered with the wayside or collateral labor of picking up matters fitted for the *kind* of traveling letters I have undertaken to write. The cause of the difficulties to which I have referred is the great and incessant occupation of the physicians of those towns, from the diseases of the Irish immigrants and the people who are compelled to have intercourse with them—a reference to which leads me to add a few paragraphs to what I said in my last.

The immigrants, in ascending the St. Lawrence, keep mostly on the Canada side, not because they prefer to be deposited in the provinces—for that is not the fact—but for the reason, chiefly, that the municipal authorities of the American towns resist their introduction. This I find is commented upon in some of the newspapers with more severity than justice or consistency, for the people of Canada regard the influx of these *fellow subjects* as a great calamity, and are afraid to come near them lest they should contract the fever. Where then is the ground for blaming the people of a neighboring nation for seeking to keep them out? If they were American paupers flying for relief from famine to Canada, it may be fairly presumed their ingress would be resisted; they would be told to look to their own country for food, and clothes, and shelter. Of the extent to which these are and will through the coming winter be furnished by the people and government of Canada, I cannot speak with precision; but all that I have seen indicates a commendable degree of consideration and humanity. When I was at Grosse Isle, one of the commissaries of supply informed me that the expenditures for that place alone were ten thousand dollars a week. When the ships arrive, all the passengers are landed, and the sick sent to the hospital sheds. After a few days, the well are sent on, nearly all at the public expense, to Quebec, and by the time they reach that city a new elimination of the sick has to be made; the others are then forwarded to Montreal, where another process of assorting is gone through, which has to be repeated even at Lachine, the port on the upper end of the island, whence they are to embark for more distant points. Of these the first is Kingston, at the head of the St. Lawrence, reached by a considerable voyage, on which many sicken even to death, and are landed by the way, especially at the intermediate town of Prescott, opposite Ogdensburg, in New York.

When, on the 7th instant, I visited the hospital sheds of that place (the same which had been erected for the Irish cholera patients in 1832), the physician, Dr. Scott, informed me that in the earlier periods of the epidemic the immigrants were sent from Montreal to Kingston in barges and schooners, where men, women and children were confined below deck, in the midst of all kinds of filth; and every vessel which came along threw upon him ten or twelve patients, some of whom would expire on the docks before he could transfer them to the sheds. Latterly, however, the transportation has been made in two small but swift steamers, and a much smaller number have sickened.

I may remark, *en passant*, that the treatment pursued by Dr. Scott, who is an experienced and respectable physician, differs considerably from that of his brethren lower down the river, as I sketched it in my last letter. Like them, he omits bloodletting, but is not afraid of emetics and cathartics. His general practice is to begin with a vomit of ipecac., and then to purge with calomel and jalap; after which he administers small doses of the mercurial until the mouth is touched. This will not happen in all cases, but when it does the patient recovers. For the intestinal irritation he gives opium alone, or combined with ipecac. and calomel. To remove topical affections, blisters are his favorite resource.

But I must return to the stream of immigration, which, continued to Kingston, requires another and more considerable depuration of the sick from the healthy. This is one of the points of the interior where many stop for permanent residence or dispersion into the country, and of course the number congregated there is large. A still greater number, however, keep on to the city where I am now writing, for its connection with the country back from the lake is much greater than that of Kingston. By the time they reach this place, they are far from the quarantine ground, and some weeks have elapsed since their disembarkation, and still the number in the hospital sheds is large. The last considerable town to which they go is Hamilton, at the head of the lake, which I shall not have time to visit.

From Grosse Isle to Toronto, it has been observed that those who go among the sick are liable to be attacked, while mingling with the well does not occasion the fever. It has also been remarked, that when individuals or families have wandered into the country and sickened, they have caused the disease in those around them. These facts seem to leave no doubt as to the generation of contagion by morbid secretion during the fever, and constitute a broad distinction between it and yellow fever.

I must pass from patients to physicians, and give you a few pages on the profession and some of the hospitals and schools of Canada. Most of the medical gentlemen whom I have seen have been educated in Great Britain and Ireland—chiefly, I think, in Edinburg—and a plurality of them are Scotchmen. In point of education, they are, I believe, superior to a corresponding number of the physicians of the United States, taken indiscriminately. That they are also superior in energy and practical tact, I am not prepared to affirm. I think it probable that in point of attainment they do not differ so much from each other as the physicians of our country. My observations, however, have been made in the towns and cities of Canada, beyond which it may be that the profession is less respectable and more diversified. In Canada East there are many French (Gallo-Canadian) physicians. I have met with a few in the cities, and found them men of science; but the greater number are in the country among what is called the *Canadian* population, and I can say nothing of their qualifications and character from personal observation. Although I have placed our brethren of Canada rather before us in point of elementary acquirements in literature and science, I have found them on the same level as to recording, digesting and publishing their experience. I met with but few who were doing their duty in those respects. Through the kindness of Dr. Fremont, of Quebec, I was furnished with a copy of the *Journal de Médecine de Quebec*, edited in 1826 and 1827 by the late Dr. Xavier Tessier, translator of Bégin's Therapeutics. In turning it over, I find that it contains reviews and analecta much more than original contributions. A much later (indeed existing) periodical, the *British American Journal of Medical and Physical Science*, edited in Montreal by Professor Hall, presents, however, a better aspect, for it has a number of able correspondents. Still, as the editor himself informed me, the mass of the physicians of Canada contribute nothing; and I have found, in my intercourse with those whom I have sought out, as in the United States, that the regular, faithful and discriminating registration of facts is practiced by a few only. As to the journal just mentioned, we shall hereafter receive it in exchange, and you will find that it is conducted with ability. Its editor, a gentleman of thorough education and great activity, is one of the professors in the medical school at Montreal; a reference to which leads me to say something on the provision made for the public education of young men in Canada. Its schools are three in number, two in the city just named, and one in that where I am now writing.

University of McGill College.— The institution bearing this unique title was founded by Mr. McGill, a wealthy and public spirited merchant of Montreal. The medical school is one of its departments. Its professors, from some technical necessity connected with the terms prescribed by the founder, are styled lecturers. They are ten in number, as follows: Anatomy, Dr. Bruneau, with Dr. Scott as Demonstrator; Surgery, Dr. Campbell; Institutes, Dr. McDonnell; Practice of Medicine, Dr. Holmes; Obstetrics, Dr. McCullough; Materia Medica and Therapeutics, Dr. Sewell; Chemistry, Dr. Hall; Clinical Medicine and Surgery, Dr. Crawford; Forensic Medicine, Dr. Fraser; Botany, Dr. Papineau. Of these the first seven lecture five times a week, the eighth twice, and the last two lecture only in the summer. Throughout the winter session, the lectures commence at 8, a. m., and, with an intermission of an hour. continue till 5, p. m. The clinical lectures are twice a week. The winter session extends from the beginning of November till the end of April, six months; the summer session commences in May, and runs through three months. One course on the institutes, medical jurisprudence, botany and practical anatomy, and two courses on all the other branches, are necessary to graduation. Before a student can be admitted to an examination, he must adduce evidence of regular attendance on the lectures, as ascertained by occasional calls of a roll; of having attended on the practice in a hospital for a year; of being twenty-one years old, and of having studied three years. The examination begins with an inquiry into his classical attainments, then comes another into his medical acquirements, conducted for an hour in private, and, finally, a defence of his thesis in public. Now, I think you will concur with me in pronouncing these requisites not only proper, but higher than those of the schools of our own country. For myself, I specially approve of the long sessions, the demand for preliminary learning, and the exacted attendance on the lectures.

School of Medicine and Surgery.—This institution is, I believe the junior of the one just described. The lectureships are Anatomy and Physiology, Dr. Nelson; Surgery, Dr. Munro; Pathology, Dr. Badgely; Materia Medica and Therapeutics, Dr. Bibaud; Obstetrics and Diseases of Women and Children, Dr. Arnoldi; Chemistry and Pharmacy, Dr. Sutherland; Practical Anatomy, Dr. Regnier. By its charter this school has not the authority to confer degrees, but by a recent arrangement the University of McGill College graduates its pupils, most of whom are Canadian French, and the lectures are exclusively in that language, as those of the other institution are entirely in the English tongue. Till this desirable compromise was effected, the professors of both schools had, I believe, to deliver their lectures in both languages. The session of this school is like that of the other, six months, and the number of lectures by each professor the same.

Medical Department of King's College, Toronto.—I have not *materiel* for a notice of this school, as from their engagements I have been unsuccessful in my efforts to see its professors, and have none of its publications.

Hospitals.—Quebec is well provided with these charitable institutions. Its[*] *Hotel Dieu* is the oldest hospital in North America, (except such as may be in Mexico) having been founded in 1637, by the Duchess d'Aiguillon, of France, and placed under the care of several nuns sent out for that purpose. Its buildings are of sufficient extent to accommodate fifty patients, and the wards are kept in excellent order.

The *General Hospital* was established by the Catholic Bishop of Quebec in 1693, is under the direction of an ecclesiastic and a number of nuns. It is rather an asylum for the old, the incurable, and the insane, than a hospital for the sick.

*Marine Hospital.*—This establishment was founded by the Canadian government, and opened for patients in 1834. It was designed for the reception of seamen and immigrants, and its funds are chiefly derived from a tax on vessels arriving, and on immigrants. It has a lecture and operating room, a museum and a library. The money received from students is applied to the purchase of books. When I visited it, I found all the wards crowded with fever patients, while hundreds were accommodated in surrounding sheds.

In addition to these, Drs. Morrin and Douglas, two of the most enterprising physicians of the city, have established a *private lunatic asylum*, which is, I believe, under the immediate care of Dr. Fremont.

In Montreal there are three charitable establishments for the sick and infirm which deserve notice:

1. *Gray Nunnery, or General Hospital of the Charitable Sisters,* founded

* Guide to Quebec.

by charity in 1692. Its object was and still is to provide permanent support and comforts for lame, infirm and superannuated persons who are destitute of means and friends. In the United States we have not, I think, an institution precisely of the same kind, for our county poor-houses should not be placed on the elevated level of this charity, and I must therefore say a word on its history. At the end of fifty-five years, it had expanded so little that it had but six inmates, its revenues were small, its debts great, and its building in a ruinous condition. From this desolation it was redeemed by the benevolent enterprise of *woman*. Miss Duffort, the daughter of a cavalry officer, married a Canadian gentleman, M. De Youville, and was left a widow at the age of twenty-eight, with a competent patrimonial estate. She resolved to devote herself to charitable objects, and meeting with some other ladies whose minds were congenial with her own, they agreed in 1737 to unite in works of charity, to live by their industry, and place their revenues in a common fund. Ten years afterwards, the establishment, in the condition just described, was transferred to them. I shall not follow them through their labors of love; suffice it to say, that at the present time, one hundred and sixty individuals are accommodated within its walls. Thus it is that woman comes to the relief of those whom our skill cannot preserve from infirmity.

2. The *Hotel Dieu* was founded in 1644, by Madame Bouillon, for the reception of the sick and diseased poor of both sexes, and up to the year 1822 was the principal place of relief for such patients. It formerly received some assistance from the French government, and latterly the provincial parliament made occasional grants for its support. It is managed by a superior and a body of nuns.

3. *General Hospital.*—Soon after the close of the last war between Great Britain and the United States, there was a great influx of poor immigrants from England, Scotland and Ireland into Montreal, which led to the formation of what was called the *Ladies' Benevolent Society*, designed expressly for their relief. From this humble yet honorable beginning arose the Montreal General Hospital. Thus all the establishments for the support and relief of the infirm poor, in that city, had their origin and impulse from that sex which is the first and last to care for the afflicted. In its progress, men of wealth came to the support of the enterprise, among whom, as standing preeminent, I may mention the Hon. John Richardson, whose name is with great justice attached to a wing of the edifice. The whole building includes nineteen wards, and has beds for a hundred and sixty patients. It is here that the students of the Montreal medical schools study clinical medicine. I visited the wards with Professor Hall, and found the medical and surgical patients mingled. The whole of *both sexes* were nursed by females

---

* Picture of Montreal.

Of the hospitals of Kingston and this city I cannot speak, not having had time to visit them, nor opportunity for acquiring a knowledge of their history and condition. I may state, however, that Toronto is the site of a public Asylum for the Insane. Hitherto they have been kept in temporary buildings, but the Canadian government, in great part from the persevering exertions of Dr. Rees, is now erecting, two miles west of the city, a capacious and magnificent edifice, which, when completed, will do honor to the province.

As we know so little of the medical profession in Canada—of its societies, of the legislation for promoting its interests, and of the associations for advancing the study of natural science, in which physicians bear so large a part—I should be tempted, had I time and the requisite information, to extend this letter much beyond the limits I have reached. On all the points to which I have referrred, our brethren of this region are much in advance of what we suppose. We ought to know more of them, and they of us. We are all European colonists from the same mother countries, and if *our political* leading strings have been severed while theirs remain, we continue in matters of literature and science to look to the other side of the great water almost as dependently as they. Then why should we not turn our faces and our hearts more inquiringly and favorably towards each other? The great lakes are common to both, and our vessels pass and repass each other every day; the physician on the banks of the St. Lawrence, when he mounts his horse to visit the sick, sees his neighbor of the opposite bank depart on the same errand; the limestone of Tennessee, Kentucky, Indiana, Ohio and New York reappears in final outcrop in Canada; the same laws of climate prevail on both sides of the boundary line, which, instead of being a mountain range, is a valley; finally, the autumnal endemics of *our* country have their feathering-out in this. These facts, it seems to me, call loudly for a more enlarged and intimate intercourse; and should what I have written contribute in the slightest degree to promote it, I shall feel that I have done some good.

Respectfully, your friend and colleague, D.

